# Development, reliability, and validity of the quality of life scale for insomnia: a health-related quality of life instrument for insomnia

**DOI:** 10.3389/fpsyt.2025.1538148

**Published:** 2025-05-16

**Authors:** Naoko Ayabe, Isa Okajima, Wataru Yamadera, Hisateru Tachimori, Hidehisa Yamashita, Naohisa Uchimura, Ken Inada, Yuichi Inoue, Kazuo Mishima

**Affiliations:** ^1^ Department of Regional Studies and Humanities, Faculty of Education and Human Studies, Akita University, Akita, Japan; ^2^ Department of Sleep-Wake Disorders, National Institute of Mental Health, National Center of Neurology and Psychiatry, Kodaira, Tokyo, Japan; ^3^ Department of Psychological Counseling, Faculty of Humanities, Tokyo Kasei University, Itabashi-ku, Tokyo, Japan; ^4^ Japan Somnology Center, Institute of Neuropsychiatry, Shibuya-ku, Tokyo, Japan; ^5^ Department of Psychiatry, The Jikei University Katsushika Medical Center, Katsushika-ku, Tokyo, Japan; ^6^ Department of Health Policy and Management, Keio University School of Medicine, Shinjuku-ku, Tokyo, Japan; ^7^ Department of Information Medicine, National Institute of Neuroscience, National Center of Neurology and Psychiatry, Kodaira, Tokyo, Japan; ^8^ Minna no Sleep and Stress care Clinic, Hiroshima, Japan; ^9^ Department of Neuropsychiatry, Kurume University School of Medicine, Kurume, Fukuoka, Japan; ^10^ Department of Psychiatry, Kitasato University School of Medicine, Sagamihara, Kanagawa, Japan; ^11^ Department of Neuropsychiatry, Akita University Graduate School of Medicine, Akita, Japan

**Keywords:** insomnia, quality of life, health-related QOL, disease-specific QOL, questionnaire

## Abstract

**Background:**

Insomnia is a quality of life (QOL) disorder complicated by various mental or physical daytime dysfunctions in addition to nocturnal insomnia symptoms. This study aimed to develop and examine the reliability and validity of a self-administered scale that can sensitively and easily assess QOL disturbances in patients with insomnia.

**Methods:**

From 122 patients with primary insomnia (mean age 53.8 ± 17.1 years), 11 items correlated with sleep-related clinical indices were extracted and designated the QOL Scale for insomnia (QOL-I). The QOL-I reliability and validity were evaluated.

**Results:**

The analysis included 93 patients with chronic insomnia (mean age 54.2 ± 16.0 years) and 228 healthy participants (45.0 ± 15.7 years). The QOL-I showed high reliability (Cronbach α=0.92). Factor analysis showed that the QOL-I has a one-factor structure. Correlation analysis between the QOL-I and other variables indicated criterion-related validity (p<0.001).

**Conclusion:**

The QOL-I demonstrated good reliability and validity and is expected to be a valuable tool for clinically assessing the QOL of patients with insomnia.

## 1 Introduction

Insomnia has an extremely high prevalence, with insomnia symptoms (1–3) and clinical criteria (4) occurring in approximately 20% and 6% of the adult population, respectively. Insomnia symptoms at night and consequent daytime impairment are the two major manifestations essential for a definitive diagnosis of insomnia (5, 6). Chronic insomnia is associated with daytime symptoms, adversely affecting social functioning and quality of life (QOL), including severe sleepiness, fatigue, memory impairment, and deteriorated attention/concentration (7–9). Daytime impairments also include behavioral changes, including the persistence of insomnia, dependence on hypnotics, and doctor shopping. However, as indicated by physiological age–related changes in sleep, if the daytime function is not impaired (i.e., QOL is maintained), the diagnostic criteria for insomnia are not fulfilled even though insomnia symptoms are observed.

The Insomnia Severity Index (ISI) (10) includes an item for measuring daytime impairment. “To what extent do you consider your sleep problem to INTERFERE with your daily functioning (e.g., daytime fatigue, ability to function at work/daily chores, concentration, memory, mood, etc.)?” In the Athens Insomnia Scale (AIS) (11), “Functioning (physical and mental) during the day” questions daytime impairment; daytime well-being and sleepiness are queried in the following items: “Sense of well-being during the day” and “Sleepiness during the day.” Health-related QOL (HRQOL) includes physical, psychological, and social factors. Daytime impairment in insomnia patients is often measured using an HRQOL scale such as the 36-item Short-Form Health Survey (SF-36) or the 8-item Short-Form Health Survey (SF-8) (12–16). The SF-36 evaluates health using eight domains: physical function, daily role function (body), body pain, overall health, vitality, social function, daily role function (spirit), and mental health. The eight domains contribute to the physical component summary (PCS) and mental component summary (MCS) scores for evaluating health status. The World Health Organization defines human health as “in a state of complete physical, mental, and social well-being and not merely the absence of disease or infirmity.” These QOL concepts are consistent with the concept of daytime dysfunction. QOL scales for patients with chronic diseases such as cancer are effective for assessing the target disease’s influence on patients’ daily lives and verifying therapeutic effects (17, 18). The SF-8 is an 8-item scale that asks questions for one item each of the eight subscales of the SF-36. However, the SF-36 and SF-8 measure overall human health; they do not specifically measure the influence of insomnia sleep problems on the QOL.

If the QOL of insomnia patients is measured on a comprehensive QOL scale, factors other than nighttime insomnia may be included. The questionnaires used to evaluate the QOL of insomnia patients include the QOL of insomnia questionnaire (19), Hotel Dieu 16 (HD-16) (20), and the Glasgow Sleep Impact Index (GSII) (21). The HD-16 is sensitive to the functional impairments experienced by patients with insomnia and has good face validity; however, the scale scores are complex to calculate and may not be easy to use in a clinical setting (22). The GSII was developed as an individualized measure specifically targeting sleep-related daytime and QOL impairments reported by patients with insomnia. This tool addresses the need for a scale that records three items relevant to each patient in their unique vocabulary and rate how impacted they were by these impairments in the past 2 weeks on a 100-mm Visual Analogue Scale (VAS), thereby capturing their individual meaning, relevance, and importance (21). Observing the clinical change before and after treatment using the VAS is optimal in patients with insomnia; however, since the GSII cannot be used to collect normative data (21), it is difficult to use it as an index to quantify and indicate the effect of the entire treatment.

Therefore, this study developed a QOL scale specialized for patients with insomnia to clarify their daytime dysfunction and examined the reliability and validity of the scale. This new scale aims to facilitate clinical evaluation by extracting daytime functional items from a standardized sleep-related scale.

## 2 Methods

### 2.1 Item developments

#### 2.1.1 Participants and settings

Participants were eligible for inclusion in the study if they were aged ≥20 years and had primary insomnia as the principal disorder based on the Diagnostic and Statistical Manual of Mental Disorders, Fourth Edition, Text Revision (DSM-IV-TR) (23) in an outpatient sleep clinic at one of four Japanese medical facilities. Ultimately, the analysis included 122 patients with an average age of 53.76 ± 17.11 years, including 61 males (average age 50.39 ± 17.48 years) and 61 females (average age 57.13 ± 16.18 years).

#### 2.1.2 Item development procedures

The Sheehan Disability Scale (SDS) (24, 25), which evaluates the degree of daytime impairment, was used as a criterion indicator to extract items related to the degree of daytime impairment due to insomnia symptoms. The SDS is a self-rated 10-point Likert scale on the degree of impairments in three items: work/school activities, family relationships, and social functioning, respectively. Higher scores indicate greater impairment. The steps for item development are as follows:

1. The correlation coefficients were calculated between the SDS total score (maximally 30 points) and each question of the following scales related to sleep problems: sleep quality (the Pittsburg Sleep Quality Index (26, 27), chronotype [the Morningness–Eveningness Questionnaire (28, 29)], stress-induced sleep reactivity [the Ford Insomnia Response to Stress Test (30)], insomnia severity [the ISI (10, 31) and AIS (11, 32)], depression [the Self-rating Depression Scale (33, 34)] and the Kessler 6-item Psychological Distress Scale (35, 36)], health-related QOL [SF-36 (16)]. To control for participants’ drug dosages, the total amount of hypnotic drugs converted to diazepam was used as a covariate.
2. Items with a correlation coefficient ≥ ± 0.3 were extracted as a guide. After extraction, multiple experts (doctors and clinical psychologists) engaged in sleep medicine consulted and excluded items judged unrelated to daytime impairment caused by insomnia. In addition, the extracted items with similar content were merged into one item, including representative content.
3. Finally, 11 items were extracted. The experts corrected them to become appropriate Japanese sentences indicating insomnia QOL (**Table 1**). Then, instructions were designed to ask for responses to the QOL-I as follows: “In the past month, how much was your state of body and mind affected due to problems with sleep? Please circle the number that most applies to you (please compare with your state of mind and body when you did not have problems with sleep).” The total score ranges from 0 to 44 points; a lower score indicates a worse QOL because of sleep. Each item was evaluated on a five-point scale, from “very (0 points)” to “not at all (4 points).”

**Table 1:** Quality of life scale for insomnia.

Item		Extremely	Very much	A little	Slightly	Not at all
1	It was difficult to make decisions about things.	0	1	2	3	4
2	It was hard to complete work, housework, or schoolwork.	0	1	2	3	4
3	Social relationships felt tiresome.	0	1	2	3	4
4	I didn’t feel motivated by work or hobbies.	0	1	2	3	4
5	I felt frustrated with trivial things.	0	1	2	3	4
6	I felt fidgety and restless.	0	1	2	3	4
7	I felt low and depressed.	0	1	2	3	4
8	I lacked energy.	0	1	2	3	4
9	Many things bothered me and I couldn’t get them out of my head.	0	1	2	3	4
10	I was easily tired.	0	1	2	3	4
11	I did not feel well (headaches, stiff shoulders, palpitations, etc.).	0	1	2	3	4

This survey asks about your sleep and your state of mind and body. Please provide answers to the questions below.

In the past month, how much was your state of body and mind affected due to problems with sleep? Please circle the number that most applies to you (please compare with your state of mind and body when you did not have problems with sleep).

The original version of the QOL-I was developed in Japanese. An English version of the QOL-I was developed and tested for language accuracy by back-translation to Japanese.

### 2.2 Reliability and validity of the QOL-I

#### 2.2.1 Participants and settings

Insomnia patients: Participants were eligible for inclusion in the study if they were aged ≥20 years and had DSM-5 (5) chronic insomnia disorder. The participants were taking hypnotics and were attending an outpatient sleep or psychiatric clinic at one of six medical facilities in Japan. They were recruited separately from the participants in the item development.

Healthy Control subjects: Participants were eligible for inclusion in the study if they were aged ≥20 years, had an ISI score <8, had no mental illness, and were not taking hypnotics. The participants were recruited using advertising media distributed throughout western Tokyo, Japan. Adult participants >20 years of age who had never engaged in shift work were eligible.

### 2.3 Data analysis and measurements

The mean value, standard deviation, floor effect, and ceiling effect were calculated for patients with insomnia. Reliability calculated the Cronbach α coefficient to test the internal consistency of the QOL-I. Construct validity was confirmed by factor analysis of the QOL-I. Criteria-related validity was evaluated by concurrent validity using correlation analysis between the QOL-I and other measurements, including the ISI (10, 31), SDS (24, 25), PCS, and MSC of SF-8 (15, 37). In addition, in patients with insomnia, a one-way analysis of variance was performed on the degree of impairment, with the ISI as an independent variable. Data were analyzed using SPSS software (version 22.0; IBM Inc., Tokyo, Japan), with the significance level set at a two-tailed level of 5%.

### 2.4 Ethical declaration

This study was approved by the Ethics Committee of the National Center of Neurology and Psychiatry and the Ethics Committees of the participating medical facilities. Patients were fully informed of the study’s purpose and content, both verbally and in writing, and they provided consent to participate voluntarily.

## 3 Results

### 3.1 Demographic data

The study analyzed 93 patients with a mean age of 54.16 ± 16.01 years, including 35 males (37.6%) and 58 females (62.4%). In the control group, 228 participants aged 44.96 ± 15.66 years were analyzed, including 95 males (41.7%) and 133 females (58.3%). The chi-squared test found no significant difference in sex between the groups (χ2 (1) =0.45, n.s.).

### 3.2 QOL-I scores for each item in insomnia patients

**Table 2** shows the item analysis results of patients with insomnia. The ceiling effect occurred in item 6, “I felt frustrated and uncomfortable,” but it was judged indispensable for daytime dysfunction by experts familiar with sleep medicine; all 11 items judged that its use was appropriate. The content validity of the QOL-I was thus confirmed through examination by multiple experts, including doctors and clinical psychologists. The distribution of the total QOL-I scores of patients with insomnia and healthy controls is provided in the **Supplementary Figure**.

**Table 2:** The score, mean values (SD), floor, and ceiling effects in insomnia patients.

Item		M	SD	Floor effects	Ceiling effects
1	It was difficult to make decisions about things.	2.44	1.14	1.30	3.58
2	It was hard to complete work, housework, or schoolwork.	2.37	1.13	1.24	3.50
3	Social relationships felt tiresome.	2.38	1.28	1.09	3.66
4	I didn’t feel motivated by work or hobbies.	2.37	1.35	1.02	3.72
5	I felt frustrated with trivial things.	2.47	1.14	1.33	3.61
6	I felt fidgety and restless.	2.95	1.16	1.79	4.10
7	I felt low and depressed.	2.37	1.19	1.18	3.55
8	I lacked energy.	2.06	1.21	0.85	3.28
9	Many things bothered me and I couldn’t get them out of my head.	2.16	1.26	0.90	3.42
10	I was easily tired.	1.57	1.16	0.41	2.73
11	I did not feel well (headaches, stiff shoulders, palpitations, etc.)	1.61	1.21	0.41	2.82

Floor effect = value ≤0; ceiling effect = value ≥4.

### 3.3 Comparison of insomnia patients and control participants

**Table 3** shows a comparison of the demographic data and measurements of insomnia patients and control participants. The Mann–Whitney U test was performed for the patients with insomnia and control participants. The QOL-I score was significantly lower in the insomnia group than in the control group (r = 0.48). In addition, the SDS, PCS, and MSC showed significant differences between the insomnia and control groups (SDS, r = 0.58; PCS, r = 0.40; MSC, r = 0.36). Although the SDS effect size was moderate, most of the control group scored 0 points, indicating a floor effect.

**Table 3:** Data on insomnia patients and healthy controls.

Parameter	Insomnia patients (*n*=93)							Healthy controls (*n*=228)							P-value	Effect size
	mean	(SD)	95% Cl.		median	mix	max	mean	(SD)	95% Cl.		median	mix	max	*p*	*r*
			lower	upper						lower	upper					
Age (y)	54.16	(16.01)	50.86	57.46	57	20	88	44.96	(15.66)	42.92	47.01	41.00	20	79	<.001a	.25
M/F (female %)	35/58 (62.4)							95/133 (58.3)							.50b	
QOL-I	24.74	(9.78)	22.73	26.76	24.00	0	44	34.96	(7.54)	33.98	35.95	37.00	6	44	<.001a	.48
ISI	14.82	(4.93)	13.80	15.83	15.00	8	28	3.54	(2.08)	3.27	3.82	3.00	0	7	<.001a	.79
SDS	11.13	(7.01)	9.69	12.57	11.00	0	30	2.81	(4.30)	2.25	3.37	1.00	0	25	<.001a	.58
PCS	43.31	(7.79)	41.71	44.91	43.92	25.04	59.94	49.60	(6.47)	48.76	50.44	50.77	21.46	61.90	<.001a	.40
MCS	43.57	(8.43)	41.83	45.31	43.56	16.57	61.28	49.44	(6.09)	48.64	50.23	50.32	26.84	62.48	<.001a	.36

M/W, male/female; SD, standard deviation; QOL-I, quality of life scale for insomnia; ISI, insomnia severity index; SDS, Sheehan disability scale; PCS, physical component summary; MCS, mental component summary.

a: Mann–Whitney U test, b: χ2 test.

Effect sizes: small (*r* = 0.1), medium (*r* = 0.3), and large (*r* ≥ 0.5).

### 3.4 Reliability (internal consistency)

Analysis of the QOL-I’s internal consistency in patients with insomnia showed a Cronbach’s alpha (range of item-deletion α) value of 0.92 (0.90–0.92).

### 3.5 Validity

To confirm construct validity, a factor analysis using the principal factor method was performed; the eigenvalue changes were 6.03, 0.99, and 0.79. The QOL-I was a one-factor model accounting for 50.55% of the total item variance; all items demonstrated loadings ≥0.50 (**Table 4**). In correlation analysis examining the concurrent criterion-related validity between the QOL-I and other variables, QOL-I correlated significantly with ISI, SDS, PCS, and MSC. The SDS and MCS correlated moderately with the QOL-I; the correlation coefficient was particularly high in patients with insomnia (**Table 5**). **Table 6** shows the results of a one-way analysis of variance for the degree of daytime dysfunction by the severity of insomnia as measured by the ISI. The QOL-I scores showed significant differences between subthreshold and moderate insomnia and between subthreshold and severity. PCS showed significant differences between subthreshold and severity. MCS showed no differences among the three groups.

**Table 4:** Factor analysis.

Item		Factor 1
8	I lacked energy.	0.87
4	I didn’t feel motivated by work or hobbies.	0.79
7	I felt low and depressed.	0.76
6	I felt fidgety and restless.	0.70
1	It was difficult to make decisions about things.	0.70
9	Many things bothered me and I couldn’t get them out of my head.	0.69
5	I felt frustrated with trivial things.	0.69
3	Social relationships felt tiresome.	0.69
2	It was hard to complete work, housework, or schoolwork.	0.68
10	I was easily tired.	0.67
11	I did not feel well (headaches, stiff shoulders, palpitations, etc.).	0.55

The principal factor method.

**Table 5:** Correlations of QOL-I with other measurements in insomnia patients and healthy controls.

Group		ISI	SDS	PCS	MCS
Insomnia	Unadjusted	-0.34**	-0.63**	0.26*	0.69**
	After controlling for age	-0.35**	-0.59**	0.31**	0.64**
Control	Unadjusted	-0.42**	-0.44**	0.13*	0.49**
	After controlling for age	-0.41**	-0.43**	0.18**	0.48**

*p < 0.05, ** *p*< 0.01 Insomnia (*n* = 93), Control *(n* = 228), QOL-I, quality of life scale for insomnia; ISI, insomnia severity index; SDS, Sheehan disability scale; PCS, physical component summary; MCS, mental component summary.

**Table 6:** Difference in degree of daytime dysfunction by ISI in insomnia patients.

Parameter	Subthreshold insomnia (*n*=43)			Moderate severity (*n*=43)			Severe insomnia (*n*=7)			*F*	*P*	Tukey HSD
	*M*	*(SD)*	95% Cl.	*M*	*(SD)*	95% Cl.	*M*	*(SD)*	95% Cl.			
QOL-I	27.65	(8.34)	25.08–30.22	22.93	(9.59)	19.98–25.88	18.00	(14.04)	5.02–30.98	4.65	0.01	I<II^†^, I<III*
SDS	7.67	(5.61)	5.95–9.40	12.88	(5.98)	11.04–14.72	21.57	(6.88)	15.21–27.93	20.4	<0.001	I<II**, I<III**, II<III**
PCS	44.99	(8.00)	42.53–47.45	42.65	(6.88)	40.53–44.77	37.03	(9.00)	28.71–45.35	3.63	0.03	I<III*
MCS	44.85	(7.85)	42.44–47.27	42.79	(8.83)	40.08–45.51	40.46	(9.32)	31.85–49.08	1.16	0.32	*n.s.*

^†^ *p* < 0.01, *p < 0.05, **p < 0.01, SD, standard deviation; QOL-I, quality of life scale for insomnia; ISI, insomnia severity index; SDS, Sheehan disability scale; PCS, physical component summary; MCS, mental component summary; HSD, honestly significant difference.

ISI score: Subthreshold insomnia (8–14), Moderate severity (15–21), and Severe (22–28).

## 4 Discussion

This study developed a convenient scale for assessing the disease-specific QOL of insomnia for measuring daytime dysfunction caused by sleep problems. The study investigated whether the QOL-I was useful as an evaluation tool for patients with insomnia.

First, the content validity of the QOL-I was confirmed by multiple experts, including doctors and clinical psychologists. Eleven items were extracted from the patients with insomnia. These 11 items were similar to depression scale items, such as the 9-item Patient Health Questionnaire (38). Insomnia is also a prodromal symptom of depression and residual insomnia (39). Therefore, items similar to the depression scale were extracted. Moreover, the study added “Please answer in comparison with the state at the time of no sleep problems.” to this scale’s teaching sentence. This sentence indicates that this scale’s purpose is to clarify the decrease in daytime dysfunction compared with before the sleep problem occurred. Furthermore, patients with insomnia complain mainly of insomnia-like dissatisfaction; many do not mention a daytime feeling of trouble. The QOL-I easily evaluates troubles focusing on daytime impairments and can indicate the ease of assessment by the therapist. The Cognitive Behavioral Therapy for Insomnia (CBT-I), considered effective for insomnia, emphasizes daily activities, including sleep hygiene. Therefore, easy assessment of functional daytime impairment and its degree will facilitate focusing on patient annoyance.

In addition, QOL-I was confirmed to have sufficient internal consistency with a high Cronbach α coefficient for patients with insomnia. “Item 11: There was a physical disorder (headache, shoulder stiffness, palpitation, etc.)” had a low value of 0.55 compared with other items, but the QOL-I has a single-factor structure, possibly related to the difference in correlation coefficients between the PCS and MCS scores. The insomnia patients’ MCS was 0.69 (0.64 after age adjustment), but the PCS was 0.26 (0.31 after age adjustment). Physical dysfunction in patients with insomnia may not be severe. Among the 11 items, the items querying the physical symptoms became the two sentences “I was tired easily” and “I was sick.” The validity of the reference was demonstrated through comparison with a healthy group. The difference between the QOL-I scores of the patients and healthy controls was approximately 10 points. Although the low number of serious patients limits the interpretation, the study found that the score decreases by approximately five points per severity increase. Although QOL items were in the ISI, no significant difference was found in the MCS scores based on insomnia severity in this study. Although this result cannot be interpreted based on the present data, no difference in QOL was found using the SF-36 between Japanese patients with and without insomnia treatment (40).

These findings suggest that the QOL of patients with insomnia may have low sensitivity. The effect size (0.48) between patients with insomnia and healthy controls was greater for QOL-I than for SF-8 (PCS, MCS), a comprehensive QOL measure. This finding suggests distinguishing healthy subjects is easier using the QOL-I score than the SF-8 score.

In contrast, the correlations between QOL-I and ISI, SDS, PCS, and MCS differed only in ISI between patients with insomnia and healthy subjects. The correlation coefficient between the ISI and QOL-I was low in patients with insomnia. Although the ISI includes QOL items, the study’s findings suggest that patients with insomnia may not necessarily show improvements in daytime functional impairment by lowering ISI scores.

Our study had several limitations. First, items on the scale were extracted from existing measures related to sleep disorders and insomnia, but criterion-related validity was evaluated using the SDS and SF-8. The evaluation might have used questionnaires that measured daytime impairment; therefore, the possibility of bias from using different insomnia patients to examine reliability and validity cannot be denied. Second, retest reliability was not assessed during the reliability review. Consequently, guaranteeing high reliability is limited, and retest reliability should be investigated further. Third, the participants were outpatients taking hypnotics at one point in time. Therefore, patients who received CBT-I were included, and the treatment dosage and duration were not controlled. Future studies should compare the clinical usefulness of QOL-I scores before and after treatment.

## 5 Conclusion

This study developed the QOL-I, an insomnia-specific QOL measure. The QOL-I demonstrated good reliability and validity and is expected to be a valuable tool for clinically assessing the QOL of patients with insomnia. In the future, clinically comparing the usefulness before and after treatment for insomnia will be necessary to measure the improvement in QOL in patients with insomnia.

## Data Availability

The raw data supporting the conclusions of this article will be made available by the authors, without undue reservation.
