# Supplementary figures and images for: Development, reliability, and validity of the quality of life scale for insomnia: a health-related quality of life instrument for insomnia
Source: Front Psychiatry. 2025 May 16;16:1538148. doi: 10.3389/fpsyt.2025.1538148 (PMC12122516; doi:10.3389/fpsyt.2025.1538148)

**Supplementary figure** Distribution of QOL-I scores in Insomnia Patients and Healthy Controls

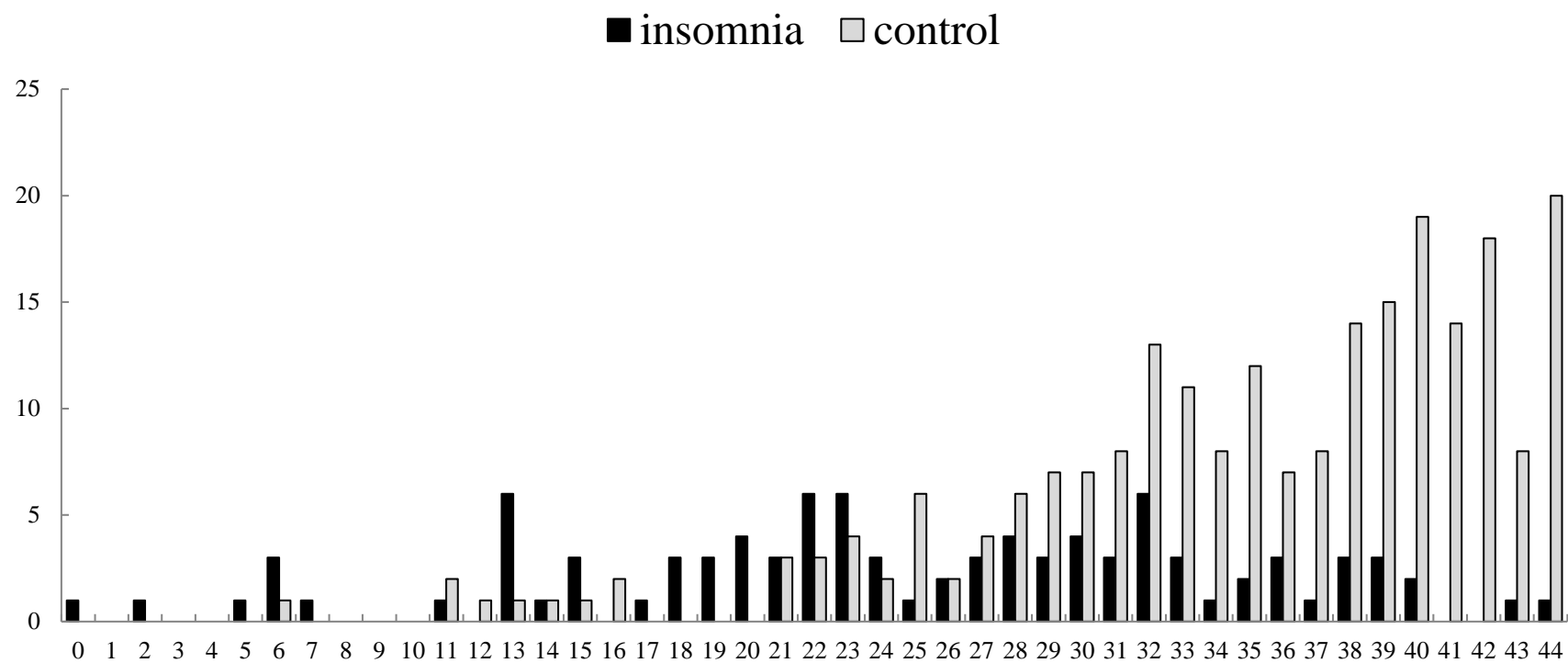

Supplement: Supplementary file 1 [file DataSheet1.pdf]
